# Optimization of 3D Immunofluorescence Analysis and Visualization Using IMARIS and MeshLab

**DOI:** 10.3390/cells12020218

**Published:** 2023-01-04

**Authors:** Zulzikry Hafiz Abu Bakar, Jean-Pierre Bellier, Wan Zurinah Wan Ngah, Daijiro Yanagisawa, Ken-ichi Mukaisho, Ikuo Tooyama

**Affiliations:** 1 Medical Innovation Research Center, Shiga University of Medical Science, Seta Tsukinowa-cho, Otsu 520-2192, Japan; 2 Brigham and Women’s Hospital, 75 Francis St., Boston, MA 02115, USA; 3 Molecular Neuroscience Research Center, Shiga University of Medical Science, Seta Tsukinowa-cho, Otsu 520-2192, Japan; 4 Department of Pathology, Shiga University of Medical Science, Seta Tsukinowa-cho, Otsu 520-2192, Japan; 5 Education Center for Medicine and Nursing, Shiga University of Medical Science, Seta Tsukinowa-cho, Otsu 520-2192, Japan

**Keywords:** IMARIS, MeshLab, colocalization analysis, 3D, decimation, simplification

## Abstract

The precision of colocalization analysis is enhanced by 3D and is potentially more accurate than 2D. Even though 3D improves the visualization of colocalization analysis, rendering a colocalization model may generate a model with numerous polygons. We developed a 3D colocalization model of FtMt/LC3 followed by simplification. Double immunofluorescence staining of FtMt and LC3 was conducted, and stacked images were acquired. We used IMARIS to render the 3D colocalization model of FtMt/LC3 and further processed it with MeshLab to decimate and generate a less complex colocalization model. We examined the available simplification algorithm using MeshLab in detail and evaluated the feasibility of each procedure in generating a model with less complexity. The quality of the simplified model was subsequently assessed. MeshLab’s available shaders were scrutinized to facilitate the spatial colocalization determination. Finally, we showed that QECD was the most effective method for reducing the polygonal complexity of the colocalization model without compromising its quality. In addition, we would recommend implementing the x-ray shader, which we found useful for visualizing colocalization. As 3D was found to be more accurate in quantifying colocalization, our study provides a novel and dependable method for rendering 3D models for colocalization analysis.

## 1. Introduction

In current cell and molecular biological research, colocalization is one of the most frequently reported visual phenomena. Findings on colocalization are used to pinpoint the precise location of the structures of interest and provide evidence for imagining their common characteristics [1]. Colocalization is crucial for analyzing metabolism, signaling processes, and transcriptional regulation [2].

The 3D approach has been increasingly used since it was initially introduced for quantifying colocalization [3]; 3D has enhanced the meticulousness of colocalization analysis and is potentially more accurate than 2D [1]. The additional axis in 3D spaces is an advantage to demonstrate a signal spatial colocalization accurately [4]. In our previous study, the implementation of 3D assists in retrieving the progression of LC3/FtMt colocalization in a patient with progressive supranuclear palsy (PSP) [5]. These particular progression patterns were unlikely to be observed by 2D, suggesting a notable advantage of 3D in perceiving colocalization. Although 3D improves our manifestation of colocalization analysis, rendering a colocalization model might generate a model with many polygons and require a particular setup to process such complex data [6,7].

Various software programs can be accessed to reduce the complexity of a 3D model [8,9]. Decimation is shown to increase a system’s efficiency in processing the model [10]; hence, it can be a valuable tool in rendering a colocalization model. We used MeshLab to decimate and render a less complex colocalization model for the analysis. MeshLab is an open-source system for manipulating and editing 3D triangular meshes. It provides a vast array of tools for altering 3D models. In the present study, we are interested in the flexibility it offers in reducing the rendering model’s complexity. When processing a 3D model, it is often necessary to reduce its geometric complexity, which is accomplished by decimation. As 3D models are made of triangle mesh, decimation is the process of reducing their number to produce a geometry with the same shape but fewer triangles [11,12]. Hence, reducing the amount of work GPU needs to render the model and significantly improving the performance. MeshLab offers different ways to simplify (decimate) triangulated surfaces, preserve geometrical detail and texture mapping, or selectively reduce the number of points in a point cloud (spatially discrete data points). It also provides different subdivision schemes, remeshing, and resampling filters to improve the geometric complexity of 3D models or to optimize point distribution and triangulation quality [13]. Furthermore, we found another study implemented a similar approach to acquire a less complex colocalization model [14]. The approach we previously described, however, was not appropriately elaborated, and the simplification procedure was empirically chosen based on the final product of the model. Although simplification can improve the efficiency of a model being processed, it might introduce a notable modification to the topology or shape of the model if implemented without careful consideration [11].

Here, we rendered a 3D model from stacked microphotography of a human brain section containing substantia nigra that underwent double immunostaining for LC3 and FtMt on immunoreactivity. Previously, we reported that the colocalization of LC3/FtMt in the nigral neurons of PSP patients revealed the progression of FtMt accumulation [5]. Our findings suggest that when a cell is subjected to an excessive amount of stress, the FtMt protective function may be overwhelmed, leading to the accumulation of mitochondrial damage. As a result, mitophagy increases, as shown by the accumulation of LC3. This ultimately leads to cell death [15].

Studies implementing MeshLab for colocalization analysis are relatively limited; therefore, its usefulness remains uncertain. In the present study, we further analyzed the accessible simplification algorithm in MeshLab and compared each procedure to assess its feasibility in generating a model with less complexity. Subsequently, the quality of the model that underwent simplification procedures was evaluated, and interpretations were made based on the criteria highlighted. Additionally, available shaders in MeshLab were scrutinized to facilitate the spatial colocalization determination.

## 2. Materials and Methods

### 2.1. Double Immunofluorescence Staining of FtMt and LC3 in the Midbrain of PSP

Double immunofluorescence staining of FtMt and LC3 was performed in the midbrain region of patients with PSP, as described in our previous study [5]. Deparaffinized sections were rehydrated in ethanol and water before being rinsed several times in 10 mM phosphate-buffered saline (PBS, pH 7.4). Deparaffinized sections were blocked with 1% hydrogen peroxide in PBS for 20 min at room temperature to inhibit endogenous peroxidase activity. The sections were then subjected to heat-induced epitope retrieval in 1 mM ethylenediaminetetraacetic acid (EDTA) for 4 min using a microwave oven. After several washings with PBS containing 0.3% Triton X-100 (PBST), tissue sections were blocked with 2% bovine serum albumin (BSA) in PBST for 30 min to reduce non-specific staining. Sections were then subjected to overnight incubation in mouse monoclonal anti-FtMt antibody (clone C65-2, 2 µg/mL) [16] and rabbit polyclonal anti-LC3 antibody (1:1000, MBL, Nagoya, Japan). After several washes with PBS, the sections were incubated for 1 h with Alexa-Fluor-555-labeled donkey anti-mouse IgG antibody (1:500, Invitrogen, A-31570, Frederick, MD, USA) and Alexa-Fluor-488-labeled goat anti-rabbit IgG antibody (1:500, Invitrogen, ab150077, Frederick, MD, USA). Following incubation, sections were rinsed with 10 mM PBS several times. True black solution (1:40 dilution in 70% ethanol, Biotium, Fremont, CA, USA) was applied for 50 s. Individually, the optimal number of z-stacks was determined for each sample (20–25 stacks with a voxel size of 0.12 µm). Digital pictures of the sections were taken using a Leica TCS SP8 confocal laser scanning microscope (Leica, Wetzlar, Germany) equipped with a Leica DMi8 microscope after coverslipping with Immunomount (Thermo Fisher Science, Ann Arbor, MI, USA).

### 2.2. 3D Rendering of FtMt and LC3 Colocalization Model

FtMt and LC3 colocalization were determined, as reported in a previous paper [4]. IMARIS 8 (v8.1, Bitplane, Belfast, Northern Ireland, UK) was used to create the 3D images run on an Intel Xeon E5-1630 v3 processor with 64 GB RAM and an AMD Firepro W5100 graphics card. TIFF images were taken with a Leica TCS SP8 confocal laser scanning microscope and were utilized to recreate the volume at full resolution. The surface rendering method was used to create the 3D models. The surface area detail level (grain size) was set at 0.24 µm, with an upper background subtraction of 0.904 µm and a lower background subtraction threshold value of 10. After processing the 3D model with rendering functions in MeshLab software (MeshLab) (v2021.07, ISTI-CNR, Pisa, Italy), the colocalization foci were highlighted. MeshLab was used to open the 3D models made with IMARIS, which were stored in the vrml2.0 format.

### 2.3. Reducing Model Complexity through Clustering Decimation (CD) and Quadric Edge Collapse Decimation (QECD)

Under the CD simplification procedure, vertices were collapsed and discretized according to the grid size (25%, 50%, and 75%, respectively) enveloping the meshes; grid size of 25%, 50%, and 75% with a world unit of 0.11479, 0.22958, and 0.34438, respectively. Under the QECD simplification, vertices were collapsed according to the desired final size of the mesh as a percentage of the initial size, which was 25%, 50%, and 75%, respectively. The quality threshold to penalize bad shape faces was set at <0.5, proportional to the model’s shape. The boundary preserving weight was set as default (1.0,) which recognized each boundary to be at the same priority and evaded the imbalanced removal of vertices. Each collapsed vertex was positioned in the quadric error-minimizing position. Non-manifold edges and vertices that appeared after each simplification procedure were removed, and eliminated faces were replaced. In addition, 30–100 edges composing the hole boundary were set at the maximum size to be closed. After the replacement, self-intersecting faces were hindered through a complete, heuristic, nondeterministic polynomial-time (NP) approach. The volume of the models was quantitated afterward.

### 2.4. Applying Shader to the Colocalization Model

A list of accessible shaders was available in MeshLab, and the ‘X-ray’ shader was appropriately suited to manifest colocalization. Ambient, edge falloff, and intensity were adjusted to enable visualization of colocalization between signals.

### 2.5. Statistical Analysis

Differences between the two unpaired groups were determined using the student’s *t*-test. Statistical significance was set at a *p*-value < 0.05. Values were expressed as mean ± standard deviation (SD). Statistical Package for the Social Sciences (SPSS version 25, IBM, Armonk, NY, USA) was used to perform the statistical analysis.

## 3. Results

### 3.1. Immunoreactivity of FtMt and LC3 in the Midbrain of PSP

**Figure 1:**
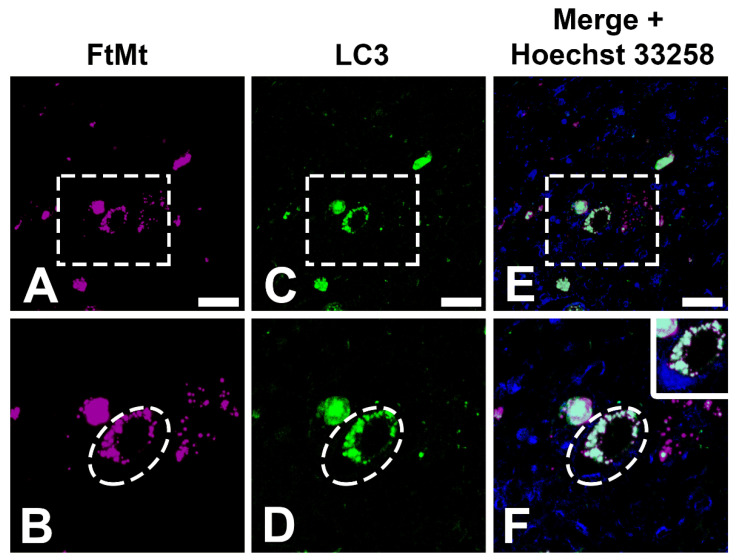
FtMt and LC3 immunoreactivity and merged images of the two immunoreactive signals. (**A**,**C**,**E**). Confocal images of FtMt, LC3, and merged signals of both immunoreactivities. There was an obvious colocalization between FtMt and LC3 immunoreactivity. (**B**,**D**,**F**). High-magnification image of the boxed area in (**A**), (**C**), and (**E**), respectively. Drawn ellipses surrounding the neuron were the acquired size for rendering a 3D model. Nuclei stained with Hoechst 33258 are shown in the merged images. Panels (**F**) contain an additional inset with vivid nuclei staining to illustrate the boundaries of the cell nucleus (magnification is the same as the main panel). All scale bars: 20 µm.

Double immunofluorescence staining, followed by confocal microscopy analysis, was performed, and immunoreactive signals for FtMt and LC3 were observed (Figure 1). As reported previously [5], FtMt and LC3 immunoreactivity was consistent here, and colocalization of the two signals was apparent (Figure 1A,C,E). Therefore, to have a fair quantitative comparison between the later implemented simplification procedure, a consistent neuron size (~25 µm) was taken for each FtMt and LC3 immunoreactive signal (Figure 1B,D,F).

### 3.2. Polygonal Number of Rendered 3D-Model after Simplification Procedure

**Figure 2:**
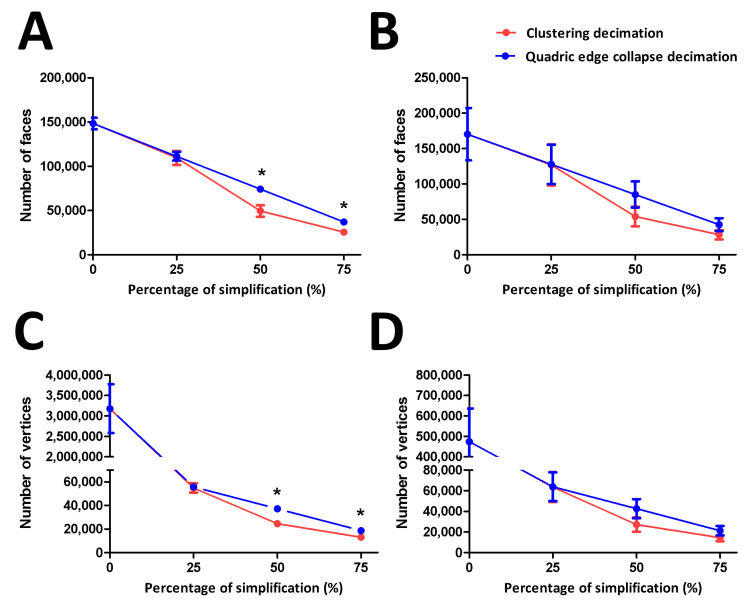
Number of faces and vertices of FtMt and LC3 3D models before and after the simplification procedure. (**A**). Number of faces of FtMt 3D models before and after 25%, 50%, and 75% simplification through CD and QECD. There was a significant decrease in the number of faces undergoing CD at 50% and 75% simplification compared to QECD. (**B**). Number of faces of LC3 3D models before and after 25%, 50%, and 75% simplification through CD and QECD. There was no significant difference in the number of faces undergoing CD at 25%, 50% and 75% simplification compared to QECD. (**C**). Number of vertices of FtMt 3D models before and after 25%, 50%, and 75% simplification through CD and QECD. There was a significant decrease in the number of faces undergoing CD at 50% and 75% simplification compared to QECD. (**D**). Number of vertices of LC3 3D models before and after 25%, 50%, and 75% simplification through CD and QECD. There was no significant difference in the number of vertices undergoing CD at 25%, 50%, and 75% simplification compared to QECD. 0% indicate the original number of faces and vertices, respectively. * demonstrates a statistically significant difference (*p* < 0.05) between CD and QECD at a particular simplification percentage. Data are presented as the mean ± standard deviation (SD).

Applying simplification to the rendered 3D model can reduce the number of faces and vertices, generating a less complex model. This procedure improves rendering performance and post-processing modifications such as texturing, shading, etc.; GPU processing time is reduced [17]. Two kinds of simplification algorithms, namely, clustering decimation (CD) and quadric edge collapse decimation (QECD), were performed on the rendered FtMt and LC3 models. The number of faces and vertices were compared in tandem (Figure 2). 

CD and QECD reduced the FtMt model to the same number of faces at a simplification level of 25%. However, compared to QECD, the CD dramatically reduced the number of faces at 50% and 75% simplification (Figure 2A). The LC3 model exhibits the same number of simplified faces for both CD and QECD at 25%, 50%, and 75% (Figure 2B). For the FtMt model, both CD and QECD reduced the number of vertices by the same amount at 25% simplification. At 50% and 75% simplification, however, the CD significantly reduced the number of vertices compared to QECD (Figure 2C). On LC3 models, CD and QECD reduced a comparable number of vertices at 25%, 50%, and 75% simplification (Figure 2D).

CD and QECD simplification algorithms could reduce a similar number of faces and vertices at 25% simplification. However, CD produced a less complex model than QECD, starting at 50% simplification on the FtMt model. However, a similar number of faces and vertices were reduced across the simplification percentage on the LC3 model. However, CD showed slightly reduced faces and vertices compared to QECD in the LC3 model. These might indicate that CD could be a preferable approach to generate a model with less complexity for further processing.

### 3.3. Effect of Simplification Procedure on the Shape of Rendered 3D Model

**Figure 3:**
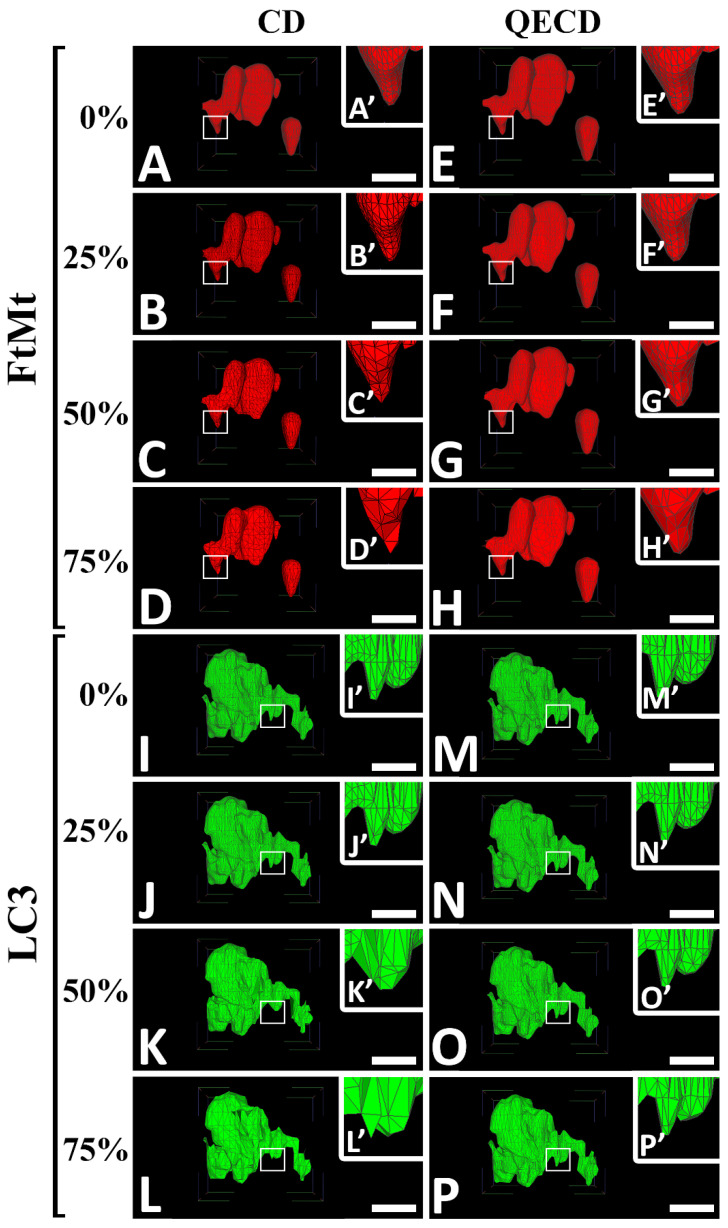
The shape of the FtMt and LC3 3D models before and after the simplification procedure. (**A**–**H**). The shape of the FtMt model at 0%, 25%, 50%, and 75% of CD and QECD simplification, respectively. (**A’**–**H’**). High-magnification image of the boxed area in (**A**–**H**), respectively. There was no notable change in the shape after undergoing 25% simplification through the CD and QECD. However, the shape was slightly changed with CD at 50% and 75% simplification. On the other hand, there was no noticeable change in the shape with QECD at 50% and 75% simplification. (**I**–**P**). The shape of the LC3 model at 0%, 25%, 50%, and 75% of CD and QECD simplification, respectively. (**I’**–**P’**). High-magnification image of the boxed area in (**I**–**P**), respectively. There was no notable change in the shape after undergoing 25% simplification through the CD and QECD. However, the shape was slightly altered with CD at 50% and 75% simplification. On the other hand, the shape did not change significantly with QECD at 50% and 75% simplification. All scale bars: 0.5 µm.

Obtaining a less complex model after simplification without introducing an artifact that can compromise the model’s integrity is preferable. Here, the shape of the post-simplified models after CD and QECD was compared to the initial rendered model and, respectively, demonstrated (Figure 3A–P’).

An accessible simplification procedure was performed to determine a plausible approach for the rendered 3D model. The shapes of the post-simplified model after the simplification procedures of clustering decimation (CD) and quadric edge collapse decimation (QECD) were put in comparison across several simplification percentages (0%, 25%, 50%, and 75%) and shown, respectively (Figure 3A–P’). There were no apparent changes in the rendered FtMt model at 25% simplification through the CD or QECD procedure (Figure 3A–F’). However, starting from 50% simplification and further, the post-simplified model by CD showed a noticeable change from the initial rendered model (Figure 3C,D’). Through the QECD procedure, there were unapparent changes to the shape of the post-simplified models up to 50% simplification (Figure 3E–G’). At 75% simplification, the shape of the models started to display a slight loss in detail (Figure 3H,H’). Similarly, the CD simplification procedure showed a noticeable change to the shape of rendered post-simplified LC3 models using CD compared to QECD. Furthermore, the shape of the post-simplified models showed no apparent differences compared to the initial rendered model (Figure 3I–J’). Starting at 50% simplification and on to the next through CD, there was an apparent alteration to the model’s shape after the simplification procedure (Figure 3K–L’). Using QECD as a simplification procedure, the shape of the post-simplified model did not exhibit apparent alteration up to 50% simplification (Figure 3M–O’). However, the shape of the post-simplified model at 75% simplification was notably altered compared to the initial rendered model (Figure 3P,P’).

Processing a 3D model with an enormous number of vertices and faces might impose on the graphics processing unit (GPU) performance. Simplifying a rendered 3D model will inevitably improve the performance of the rendition process; however, it may cause a loss in detail or introduce a topological noise to the 3D model. Hence, selecting an appropriate simplification procedure will enhance the performance of the rendition while maintaining the shape of the post-rendered model from notable alteration-induced noises. We found that CD imposed a considerable change to the shape of the rendered FtMt and LC3 models, starting at 50% simplification. In contrast, QECD exhibited a minor alteration up to 50% simplification in the shape of the post-simplified models. These might suggest that through the QECD procedure, a post-simplified model with a minimal polygonal number could be achieved without compromising the quality of the model.

### 3.4. Quantitative Analysis of the Rendered Model Relative to Simplification Procedure

**Figure 4:**
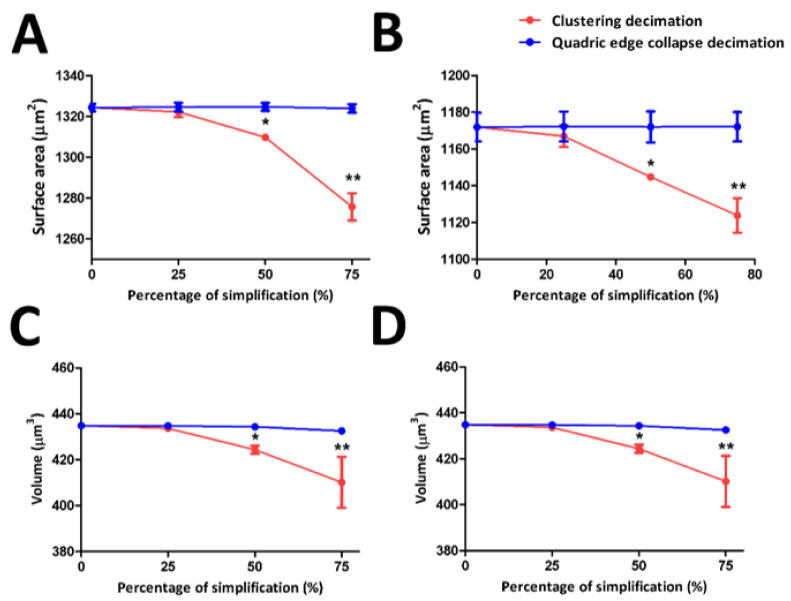
The surface area and volume of FtMt and LC3 3D models before and after simplification. (**A**,**B**). Surface area of FtMt and LC3 3D models before and after 25%, 50%, and 75% simplification through CD and QECD, respectively. There were no significant changes in the surface area of the FtMt model at 25% simplification through CD compared to the original model. However, there were significant changes in the volume of the model at 50% and 75% simplification compared to the initial volume of the FtMt and LC3 models, respectively. (**C**,**D**). Volume of the FtMt and LC3 3D models before and following 25%, 50%, and 75% simplification by CD and QECD, respectively. Comparing the FtMt model with a 25% simplification through CD to the original model, there were no significant changes in surface area. Nonetheless, the volume of the model at 50% and 75% simplification differed significantly from the initial volume of the FtMt and LC3 models, respectively. 0% indicates the original number of faces and vertices, respectively. * indicates a significant difference (*p* < 0.05) at 50% simplification compared to the initial rendered model (0%). ** indicates a significant difference (*p* < 0.05) at 75% simplification compared to the initial rendered model (0%). Data are presented as the mean ± standard deviation (SD).

The surface areas and volumes of FtMt and LC3 rendered 3D models prior to and following 25%, 50%, and 75% simplification by CD and QECD were demonstrated. (Figure 4). 

The surface areas of FtMt and LC3 rendered 3D-models before and after 25%, 50%, and 75% simplification through CD and QECD were shown, respectively (Figure 4A,B). There was no significant change in the surface area of the post-simplified FtMt models with CD at 25% simplification. However, at 50% and 75% simplification, the volume was significantly reduced compared to the initially rendered FtMt model. Similarly, shown with the LC3 model through CD, at 25% simplification, there was no apparent change in the volume. However, at 50% and 75% simplification, the volume was significantly reduced compared to the initially rendered LC3 model, as opposed to QECD, where there was no noticeable change in the surface area throughout the simplification percentage in FtMt and LC3 model compared to the initial rendered model. The rendered 3D models of FtMt and LC3 before and after 25%, 50%, and 75% simplification by CD and QECD are shown, respectively (Figure 4C,D). The volume of the post-simplified FtMt models with CD at 25% simplification did not change significantly. However, the volume was significantly reduced at 50% and 75% simplification compared to the initially rendered FtMt model. Similar to the LC3 model through CD at 25% simplification, there was no discernible volume change. At 50% and 75% simplification, the volume of the LC3 model was significantly reduced compared to the initial rendering. In contrast to QECD, the volume of the FtMt and LC3 models did not appear to change as the percentage of simplification increased compared to the initial rendered model.

Selecting the appropriate simplification procedure is essential to reduce the models’ complexity while ensuring that the model’s quality is not compromised. Based on the criteria measured, QECD simplification is preferable to CD as it retains the quality of the post-simplified FtMt and LC3 from notable alteration relative to the initial rendered model up to 50% simplification.

### 3.5. Implementation of Shader to the Colocalization Model

**Figure 5:**
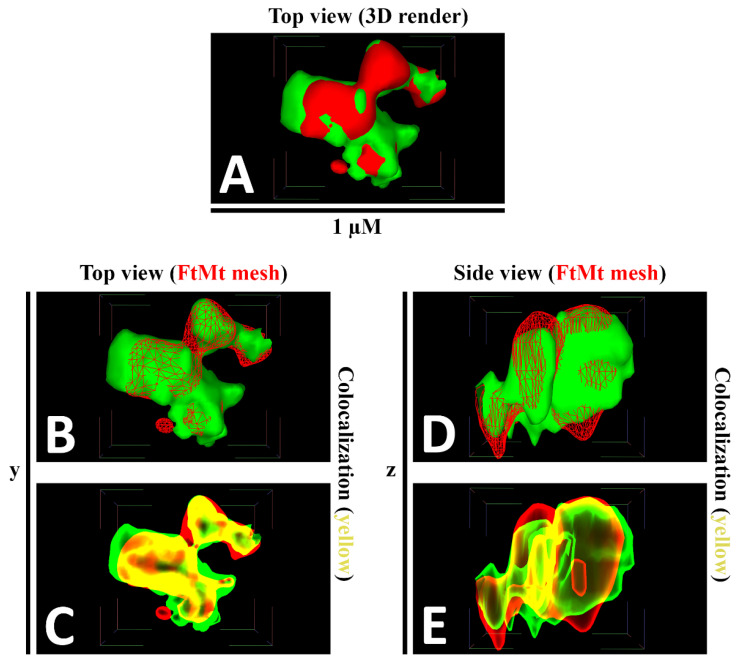
The FtMt and LC3 colocalization model is rendered using a shader. (**A**). Colocalization model before administrating the shader. Colocalized region was not visible as it was obstructed by the respective rendered FtMt and LC3 3D model. (**B**). Top view of FtMt and LC3 colocalization model without application of shader. FtMt signal was presented as a network/mesh while LC3 signal was presented as a solid surface. (**C**). Top view of FtMt and LC3 colocalization model with shader applied. Colocalization region between FtMt and LC3 signal was visible within the model. (**D**). Side view of FtMt and LC3 colocalization model. FtMt signal was presented as a network/mesh while LC3 signal was presented as a solid surface. (**E**). Side view of FtMt and LC3 colocalization model without application of shader. FtMt signal was presented as a network/mesh while LC3 signal was presented as a solid surface. Colocalization region between FtMt and LC3 signal was visible within the model.

A shader was applied to facilitate the presentation of colocalized meshes in a rendered 3D model. Various shaders were accessible in the MeshLab, but subsequently, the X-ray shader was found to be the most appropriate approach for our observation. Here, an X-ray shader was applied to the FtMt and LC3 colocalization model and demonstrated, respectively (Figure 5A–E). In the absence of a shader, the spatial localization of each mesh was unable to be presented appropriately, which affected the precise presentation of the colocalization (Figure 5A,B,D). However, with the X-ray shader, the opacity of the meshes could be adjusted, and colocalization between both meshes could be observed and presented (Figure 5C,E).

## 4. Discussion

In our previous study, we implemented a 3D analysis to trace the spatial localization of FtMt and LC3 immunofluorescence signals and further ascertain their colocalization in nigral neurons of patients with progressive supranuclear palsy [5]. However, we did not comprehensively scrutinize our approach using 3D, which leaves a gap in our means of analysis. In this study, we extensively analyzed our novel approach of using MeshLab to achieve an appropriate colocalization model, as we previously reported. Before the other process can be implemented, it is essential to identify the finest setting in IMARIS to obtain a precise justification for the difference between the simplification procedures in MeshLab. In order to generate an accurate 3D model that closely resembles the original image of the markers, it is essential to pay close attention to the threshold setting, which affects the precision of surface reconstruction. The default setting allows the threshold to be assigned either automatically or manually; however, because the signal quality varies between samples, it has to be modified accordingly [18].

**Figure 6:**
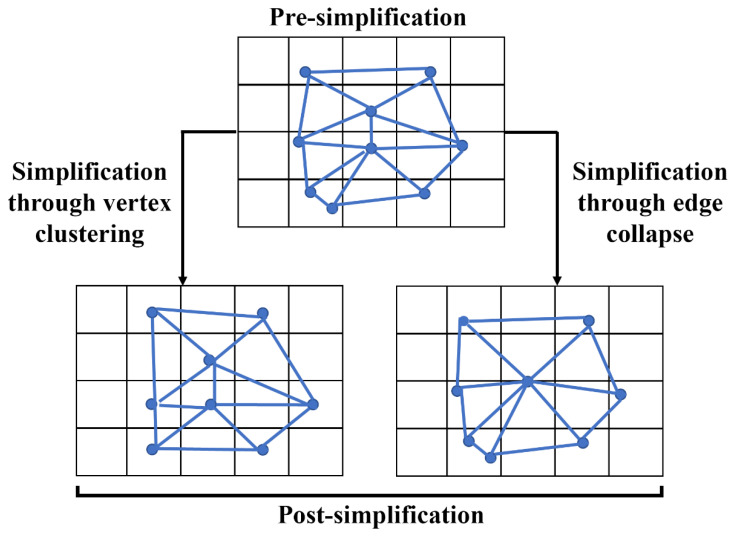
Illustration of a model that underwent vertex clustering and edge collapse simplification. Although both approaches produce fewer vertices than the initial model, clustering decimation was likely to introduce changes to the model’s shape. Whereas the model underwent edge collapse, simplification appeared to retain its shape.

Before generating a 3D colocalization model, we found that selecting an appropriate simplification procedure is essential to acquiring a less complex model for further processing. Here, CD and QECD algorithms were compared, demonstrating that CD could generate a less complex model with a slightly reduced polygonal number compared to QECD. To the best of our knowledge, this study was the first to report a comparison between CD and QECD in MeshLab, and from our analysis, the capability of CD to produce a slightly lesser number of vertices than QECD was anticipated. The approach of determining the representative vertex might be the stem of the differences between the number of vertices decimated by CD or QECD. The representative vertex in CD was determined by averaging contained vertices within the same grid, while in QECD, adjacent vertices were collapsed together to produce a newly formed vertex [6]; hence, lesser vertices were decimated compared to CD. We provide a visual difference in creating representative vertex for both approaches (Figure 6). Although CD produced a slightly less complex model compared to QECD, averaging the vertices to determine representative vertices distorted the model’s shape [19]. These differences may suggest that the quality of the post-simplified model must be considered to ensure that the exact interpretation of a colocalization model following the simplification procedure does not diverge [20].

In this study, we further examined the quality of the post-simplified model that underwent CD or QECD. We observed a significant change to the model’s shape after CD while we obtained a notable simplified model using QECD without compromising the shape from the initial rendered model. Differing from CD, the quadric error metric (QEM) was incorporated in QECD in determining the representative vertex for a newly generated simplified model [21]. QEM has been widely embedded in various similar-mean software, e.g., visualization toolkit (VTK), and our observation was in sync with the previous study [22]. Although a few studies have shown that QEM alone does not retain the original shape of the model [19,23], we did not, however, observe any shape deformation in the post-simplified model up to 75% simplification through QECD. Nonetheless, our analysis did not further characterize the shape beyond the observed simplification percentage. Hence this may suggest that QECD could preserve the model’s shape until a specific limit of simplification. Further highlighting the potential of the implemented simplification procedure, we performed quantitative analysis on the surface area and volume of the post-simplified model. CD notably reduced the surface area and volume of the post-simplified model, which was conversely shown by QECD. Simply averaging the mean for representative vertex was shown to rarely yield a satisfying simplified model [7,19] as indicated in CD. However, implementation of QEM was previously shown to maintain the geometrical characteristics property of the post-simplified model [22,24], which agrees with our findings. Although there is a growing improvement to the QEM approach [23,25], still, incorporation of QEM in QECD proves to have a promising potential to generate a simplified model without compromising the quality and integrity of the model.

Our study took advantage of the accessible shaders in MeshLab and produced a suitable shader to visualize colocalization. As reported previously, we were able to retrace the pattern of FtMt and LC3 colocalization assisted with the X-ray shader [5]. We found that at the latest colocalization pattern, LC3 was thoroughly encapsulated within FtMt. There was a limitation to rendering such colocalization using IMARIS, which was made possible through the X-ray shader in MeshLab. Additionally, features available in the X-ray shader provide a number of flexibilities to the user to highlight the exact colocalization between the signals.

In conclusion, this study optimizes our previous approach to using 3D for colocalization analysis with MeshLab (ISTI-CNR, Pisa, Italy). By comparing the simplification algorithm, we found that QECD was best to reduce the polygonal complexity of the colocalization model without compromising the quality after simplification. Furthermore, we suggest the implementation of the X-ray shader, which was found useful for visualizing colocalization. A flowchart that outlines our current protocol was provided for the user’s convenience in replicating our method (Supplementary Figure S1). Finally, as 3D was found to be more accurate in quantifying colocalization [1], our study provides a novel and reliable approach to render a 3D model for colocalization analysis.

## Data Availability

Not applicable.

## Supplementary Materials

The following supporting information can be downloaded at: https://www.mdpi.com/article/10.3390/cells12020218/s1. Figure S1: Flow chart of our present protocol.
