# Supplementary material for: Optimization of 3D Immunofluorescence Analysis and Visualization Using IMARIS and MeshLab
Source: Cells. 2023 Jan 4;12(2):218. doi: 10.3390/cells12020218 (PMC9856541; doi:10.3390/cells12020218)
Supplement: Supplementary file 1 [file cells-12-00218-s001.zip › cells-2065814-supplementary.pdf]

cells

Supplementary Information for

Optimization of 3D Immunofluorescence Analysis and Visualization using IMARIS and MeshLab

Zulzikry Hafiz Abu Bakar [1], Jean-Pierre Bellier [2], Wan Zurinah Wan Ngah [1], Daijiro Yanagisawa [3], Ken-ichi Mukaisho [4,5], and Ikuo Tooyama [1,*]

[1] Medical Innovation Research Center, Shiga University of Medical Science, Seta Tsukinowa-cho, Otsu 520-2192, Japan
[2] Brigham and Women's Hospital, 75 Francis St., Boston, MA 02115, USA
[3] Molecular Neuroscience Research Center, Shiga University of Medical Science, Seta Tsukinowa-cho, Otsu 520-2192, Japan
[4] Department of Pathology, Shiga University of Medical Science, Seta Tsukinowa-cho, Otsu 520-2192, Japan
[5] Education Center for Medicine and Nursing, Shiga University of Medical Science, Seta Tsukinowa-cho, Otsu 520-2192, Japan
* Correspondence: kinchan@belle.shiga-med.ac.jp

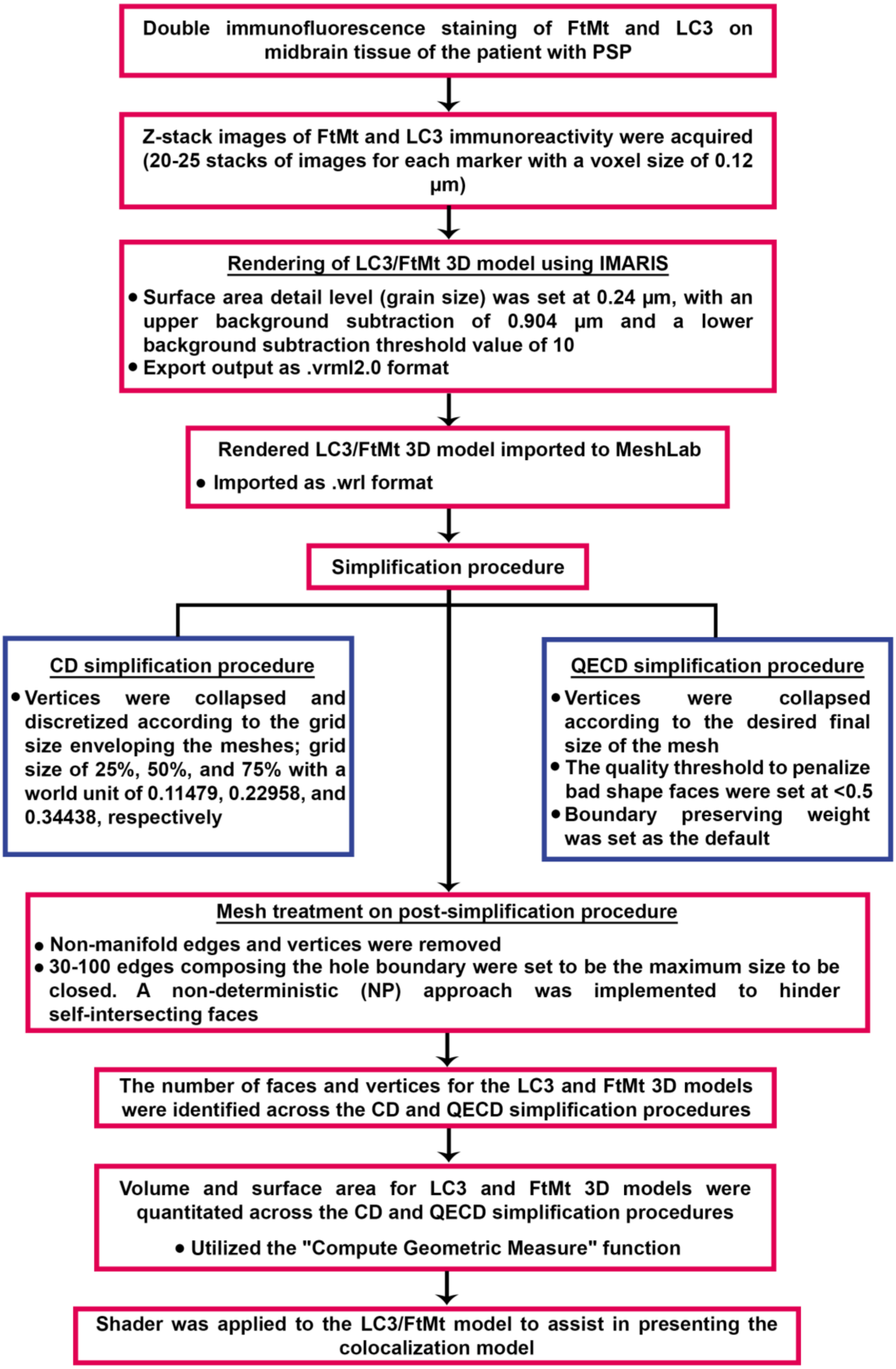

Figure S1. Flowchart of the current protocol to analyze and visualize 3D immunofluorescence signal using IMARIS and MeshLab. IMARIS 8 (v8.1, Bitplane) was utilized to render the 3D model with the specified parameters. IMARIS generated. vrml2.0 as the output format and converted it to.wrl in order to be

compatible with MeshLab (v2021.07), which was used to perform subsequent simplification procedures. The simplification procedure may generate non-manifold edges and vertices, hindering the capability to quantify the 3D model. Consequently, some protocols were put into place to obtain a simplified 3D model devoid of these features. An X-ray shader was applied to facilitate the visualization of immunofluorescence signal colocalization.
